# Right external globus pallidus changes are associated with altered causal awareness in youth with depression

**DOI:** 10.1038/tp.2015.148

**Published:** 2015-10-06

**Authors:** K R Griffiths, J Lagopoulos, D F Hermens, I B Hickie, B W Balleine

**Affiliations:** 1 Behavioural Neuroscience Laboratory, Brain & Mind Research Institute, University of Sydney, Camperdown, NSW, Australia

## Abstract

Cognitive impairment is a functionally disabling feature of depression contributing to maladaptive decision-making, a loss of behavioral control and an increased disease burden. The ability to calculate the causal efficacy of ones actions in achieving specific goals is critical to normal decision-making and, in this study, we combined voxel-based morphometry (VBM), shape analysis and diffusion tensor tractography to investigate the relationship between cortical–basal ganglia structural integrity and such causal awareness in 43 young subjects with depression and 21 demographically similar healthy controls. Volumetric analysis determined a relationship between right pallidal size and sensitivity to the causal status of specific actions. More specifically, shape analysis identified dorsolateral surface vertices where an inward location was correlated with reduced levels of causal awareness. Probabilistic tractography revealed that affected parts of the pallidum were primarily connected with the striatum, dorsal thalamus and hippocampus. VBM did not reveal any whole-brain gray matter regions that correlated with causal awareness. We conclude that volumetric reduction within the indirect pathway involving the right dorsolateral pallidum is associated with reduced awareness of the causal efficacy of goal-directed actions in young depressed individuals. This causal awareness task allows for the identification of a functionally and biologically relevant subgroup to which more targeted cognitive interventions could be applied, potentially enhancing the long-term outcomes for these individuals.

## Introduction

Depression is the leading cause of disability and morbidity in adolescence and young adulthood, with prevalence rates as high as 15% by the time a person reaches 25 years of age.^1, 2^ With its peak age of onset occurring between 15 and 29 years,^1^ depression often occurs at a critical time for social, academic and occupational functioning and development.^3^

Although low mood and anhedonia are core features of depression, cognitive dysfunction is another fundamental aspect of the disorder. Furthermore, greater cognitive impairment is predictive of poorer academic, occupational and daily functioning independent of mood severity and is also associated with higher rates of recurrent illness.^4^ The cognitive symptoms of depression include deficits in working memory, learning and executive functioning,^5, 6, 7^ which have been shown to persist even after mood symptoms resolve.^8^ Importantly, aspects of executive function have long been recognized as depending on the accuracy of causal judgments and particularly, with regard to goal-directed action, on judgments concerning the causal efficacy of actions and their specific consequences.^9, 10^ This is particularly true both of the ability to exert control over one's actions and to perceive the effects of actions on the environment, both of which have been thought deficient in depression,^10, 11, 12, 13^ and that, in the context of action control, are necessary for selecting appropriate actions and so for effective decision-making.^11^ Previous studies have, however, provided mixed evidence as to whether people with depression experience deficits in causal awareness (for example, see Alloy and Abramson,^12^ Eshel and Roiser^13^ and Pizzagalli *et al.*^14^), likely due to heterogeneity in the measures employed and within the disorder itself.^15^ Here we focused on action-related causal judgments in depression examining variations in the relationship between those judgments and the neural processes that support them.

Functional imaging studies in healthy subjects have demonstrated that encoding specific action-outcome contingencies is mediated by a cortical–basal ganglia circuit primarily involving a projection from the medial prefrontal cortex to the anterior region of the caudate nucleus.^16, 17^ Functional and structural abnormalities are commonly reported within these two regions in depression,^18, 19, 20, 21, 22^ lending support to the hypothesis that any deficit in causal awareness in depression stems from changes in this circuit. There is, however, also evidence of reduced structural volumes within a cortical–striatal–pallidal–thalamic brain circuit, not only in structures critical for cortical regulation of the basal ganglia but also in those that mediate feedback from the basal ganglia, particularly the pallidum, to regulate cortical function.^23^

Despite this evidence, no previous study has directly investigated the relationship between causal awareness and brain structural integrity in a young depressed sample. Identification of functionally relevant structural changes within these early stages of illness may be critical for identifying those who would benefit from targeted cognitive therapies that could change the trajectory of illness. Here we used a probabilistic choice task in which we varied action-outcome contingencies in order to assess changes in the subjects' causal judgments regarding these actions. This measure of causal awareness was then correlated with whole-brain and subcortical gray matter volumes. To further investigate the relationship between volumetrics and causal awareness, shape analysis was employed to determine the location and pattern of structural changes.^24^ Combined with probabilistic tractography, these measures enabled the identification of regionally localized atrophy and allowed us to elucidate a specific circuit correlating with changes in causal awareness in young people with depression centered on the external globus pallidus.

## Materials and methods

### Subjects

Forty-three subjects with unipolar depression (DEP) aged 16–29 years were recruited from the Youth Mental Health Clinic, Brain and Mind Research Institute, and Headspace, Sydney, Australia,^25^ and 21 demographically similar healthy controls (HCs) were recruited from the surrounding community (see Table 1). Exclusion criteria for both clinical and control groups were history of neurological disease (for example, head trauma and epilepsy), intellectual and/or developmental disability and insufficient English for neuropsychological assessment.

Controls were screened for psychopathology via clinical interview and patients were tested under ‘treatment-as-usual' conditions. The experiment was conducted in a single replication. All participants provided written informed consent and the study was approved by the University of Sydney Human Research Ethics Committee.

### Clinical and neuropsychological assessment

All participants underwent clinical and neuropsychological assessment as previously reported.^25^ Patients were determined to have a primary diagnosis of DEP by a psychiatrist, according to Diagnostic and Statistical Manual of Mental Disorders, 4th edition, Text Revision criteria.^26^ To quantify current depressive symptoms, a research psychologist made clinical ratings using the Hamilton Depression Rating Scale (17 items)^27^ and also rated participants on the Social and Occupational Functioning Assessment Scale.^28^ Premorbid intelligence (predicted intelligence quotient) was estimated based on performance on the Wechsler Test of Adult Reading.^29^

### Causal awareness task

**Figure 1:**
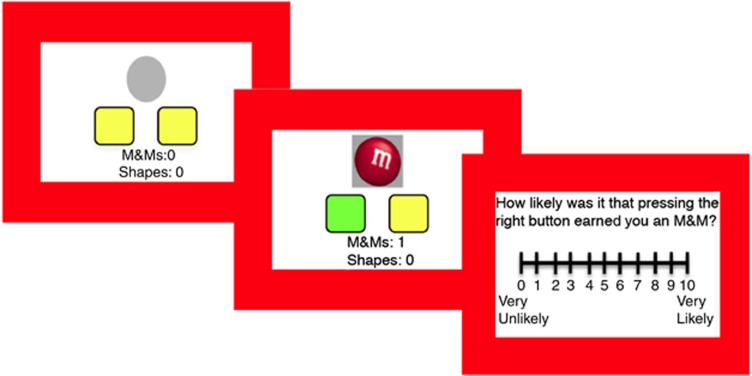
Experimental task. Participants were able to choose between two buttons, to maximize reward. Unsuccessful button presses were signaled by a gray circle, whereas rewarded responses were signaled by a 1000-ms reward stimulus presentation, and the responsible button was highlighted in green. After each block of trials, participants rated how causal each button was.

Subjects performed a self-paced instrumental learning task in which they chose between two actions (left or right button press) in order to maximize food rewards (M&M chocolates or BBQ-flavored crackers). Two yellow boxes on the computer screen corresponded to two yellow keys available on a keyboard (see Figure 1, and participant instructions in the Supplementary Materials). During each of the twelve 40-s blocks, reward probability was always higher on one action (A_HIGH_) than the other action (A_LOW_). Across blocks, A_HIGH_ switched location (left or right), and the reward probability varied (0.25, 0.125, 0.08), and as the reward probability of A_LOW_ was set at 0.05, this altered the relative difference in the contingency between actions. No predictive cues were presented on the screen; our aim was to reduce interference from Pavlovian learning processes. On non-rewarded responses, a gray circle appeared in the center of the screen for 250 ms, whereas during rewarded responses the rewarded key turned green and an image of the food reward appeared in the center of the screen for 500 ms. A tally of accumulated winnings remained on the bottom of the screen for the duration of the task. At the end of each block, participants were asked to make causal judgments, that is, to judge, on a 10-point Likert scale, how likely it was that pressing each button earned them rewards on the previous trial (0, not at all likely, 10, extremely likely). The task began with a 0.25-contingency practice block, a hunger rating (0, not hungry at all, to 10, extremely hungry) and a pleasantness rating for each food outcome (−5, not at all pleasant, to +5, extremely pleasant). Choice performance and causal awareness were computed by averaging the proportion of optimal key choices and optimal key ratings (A_HIGH_/A_HIGH_+A_LOW_) from the three contingency conditions.

Clinical and behavioral statistical analyses were performed using IBM SPSS Version 20 (SPSS, Chicago, IL, USA). Analyses of variance and *χ*^2^-tests were used to examine group differences in demographics, clinical information and contingency task performance. Assumptions of normality were assessed using Kolmogorov–Smirnov tests, and Mann–Whitney *U*-tests were used to examine group differences in variables with non-normal distribution. Equality of variance was assessed using Levene's test. Differences between groups on behavioral measures were tested using multivariate analyses of variance, with age and education as covariates. Pearson correlations were conducted between task variables and clinical and quality of life scales. Alpha levels were set at 0.05 (two tailed).

### Imaging

See Supplementary Materials for image acquisition details. FMRIB Software Library tools (www.fmrib.ox.ac.uk) were used in all imaging analyses (version 5.0.1).

### Voxel-based morphometry

Voxel-based morphometry (VBM) used standard methodology (see Supplementary Methods). Briefly, we used an unbiased optimized VBM protocol^30^ before all T1-weighted images were transformed into standard space using a limited degrees-of-freedom nonlinear model to ensure spatial alignment and images were corrected for nonuniformity. After tissue-type segmentation and gray matter alignment a study-specific averaged template was created, to which gray matter partial volume images were re-registered. Segmented images were smoothed and correlations between causal awareness and gray matter volumes were assessed using permutation-based general linear models, both with and without co-variance for depression severity. Group differences were assessed using *F*-statistics co-varied for age and education.

### Volumetrics

The semi-automated FIRST^24^ routine was used to segment the following: bilateral caudate nucleus, putamen, pallidum and thalamus. Segmentations were visually inspected to ensure that there were no gross registration or segmentation errors. Tissue-type segmentation carried out using FAST4 was used to calculate intracranial volumes, which were used to correct for differences in head size. The aforementioned subcortical region volumes were corrected for intracranial volume variation so as to provide a common space for cross-sectional morphometric comparisons. All statistical analyses were conducted using IBM SPSS statistics. Pearson correlations were conducted within each group between causal awareness and subcortical volumes, and clinical measure scores. Volumetric differences between groups were also assessed. The alpha level for volume analyses was set at a very conservative Bonferroni-corrected *P*<0.006 (*P*<0.05 divided by eight regions of interest).

### Shape analysis

Localized shape differences in subcortical regions were examined in cases where volumetric changes were significantly correlated to the task performance. Correlations between causal awareness and subcortical region shape were assessed within the depressed group on a per-vertex basis using permutation-based general linear models, both with and without co-variance for depression severity and volume of the structure. Group differences were also assessed using *F-*statistics co-varied for age and gender (see Supplementary Methods for full details).

### Diffusion tensor imaging preprocessing and qualitative probabilistic tractography

Diffusion probabilistic tractography was run for each subject from a seed mask of vertices significantly correlated with causal awareness within the depressed group. Tractography was performed from every voxel within the seed mask to build up a connectivity distribution. We fitted a three-fiber orientation diffusion model^31^ to estimate probability distributions on the direction of fiber populations at each brain voxel in the diffusion space of each subject. To visualize tracts efferent and afferent to the seed mask, individual's three-dimensional files were thresholded to the top 1% of tracts and binarized, before being combined into a group image (see Supplementary Methods for full details).

## Results

### Demographics and clinical information

The demographic, clinical and task motivational characteristics of participants are shown in Table 1. Groups did not differ significantly with regard to age, gender, education attainment, predicted intelligence quotient or any task motivational measures. Mann–Whitney *U*-tests indicated that DEP had increased DEP severity and reduced social and occupational functioning relative to CON, *P*<0.001.

### Causal awareness was reduced in depressed subjects independently of mood severity

**Figure 2:**
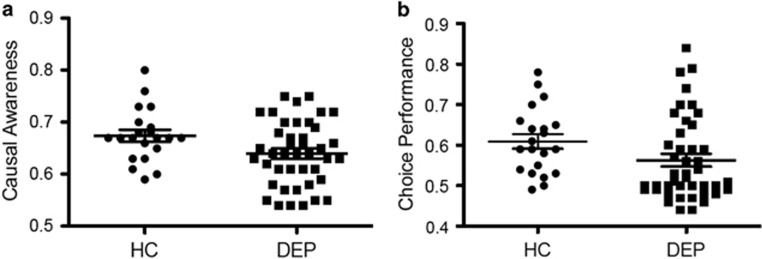
Group behavioral differences. (**a**) Healthy controls made a significantly higher proportion of optimal action ratings than DEP, *P*=0.02 (**b**). However, although marginal (*P*=0.08), there was no statistical difference between groups in proportion of optimal choices. Mean and s.e.m. are indicated for each group. DEP, depression; HC, healthy control.

**Figure 3:**
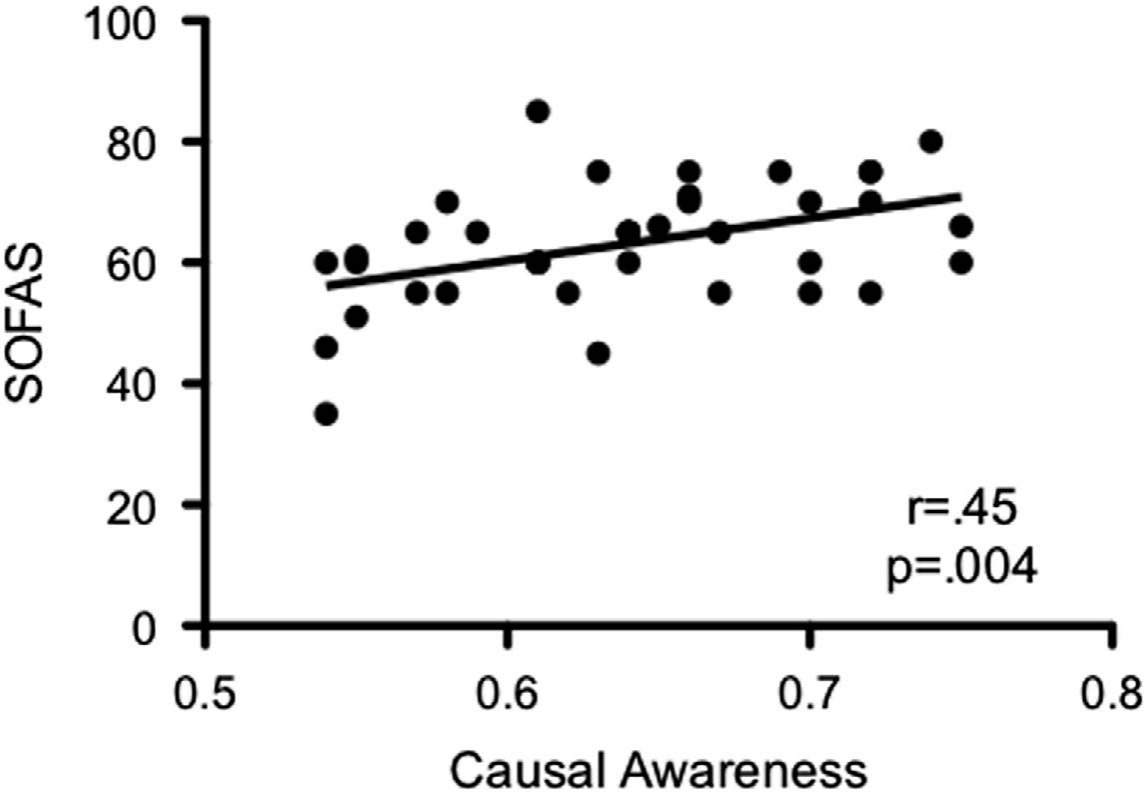
Functional relevance. Positive correlation between causal awareness and clinician-rated SOFAS scores in youth with depression. SOFAS, Social and Occupational Functioning Assessment Scale.

Our primary measure of interest was the information that the subjects drew from their behavioral experience as measured by their causal judgments. Importantly, at a group level, DEP (*m*=0.63, s.d.=0.07) had significantly reduced causal judgments relative to HC (*m*=0.67, s.d.=0.05), *t*(62)=2.35, *P*=0.022 (Figure 2a). Although there were no correlations between causal awareness and DEP severity measures in the clinical group, there was a strong positive correlation between causal awareness and Social and Occupational Functioning Assessment Scale scores, *r*=0.45, *P*=0.004 (Figure 3), indicating that individuals with reduced causal awareness demonstrated poorer social and occupational functioning. There was a statistical trend for a small negative correlation between DEP severity and social and occupational functioning, *r*=−0.29, *P*=0.07. Although trending toward a difference, behavioral performance did not differ significantly between the HC and DEP groups (*P*=0.08—Figure 2b).

### Causal awareness predicted right pallidal size only in depressed subjects

**Figure 4:**
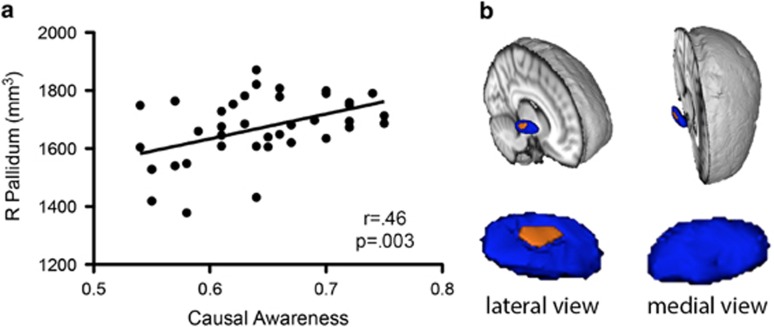
Causal awareness and subcortical volume and shape in depressed subjects. (**a**) Positive correlation between contingency awareness and volume of the right pallidum (**b**). Vertex-wise shape analysis. Average shape of the right pallidum in depressed subjects (blue), with orange regions representing surfaces where decreases in causal awareness correlate with an inward location of vertices. Total pallidum size was accounted for as a covariate of no interest. Top image shows varying rotations of a Montreal Neurological Institute template brain, with the right hemisphere cut away to reveal the three-dimensional mesh of the pallidum. Bottom images show lateral and medial views of the right pallidum.

T1-weighted images were not obtained from four depressed participants due to scanning artifacts or non-completion of the imaging component. There was a positive relationship within the DEP group between contingency awareness and volume (mm^3^) of the right pallidum, *r*=0.46, *P*=0.003—see Figure 4a, which survived a stringent Bonferonni correction (0.05 partitioned over 8 regions of interest, *P*=0.006). In order to determine whether other factors moderated these relationships, age, Social and Occupational Functioning Assessment Scale score, Hamilton Depression Rating Scale score and causal awareness were entered into stepwise multiple regression analyses to predict right pallidal size. In predicting pallidal size, only the causal awareness correlation remained significant (*P*=0.003), and was the sole variable in the significant prediction model, *F*(1,37)=10.01, *P*=0.003. Causal awareness accounted for ~22% of the variance in right pallidal size.

There was no correlation between causal awareness and any region-of-interest volumes in the HC group, suggesting that a smaller right pallidum alone does not necessarily result in poorer causal awareness. To investigate whether causal awareness-related volume variance in DEP occurred in specific regions of the right pallidum, we subsequently conducted vertex-based shape analyses.

### Reduced causal awareness correlated with inward location of vertices on the dorsolateral aspects of the right pallidum

Comparison of vertex locations in the depressed group showed a significant correlation between reduced causal awareness and a significant inward location of vertices on the dorsolateral aspect of the right pallidum (Figure 4b). This finding held when DEP severity, age and total pallidum volume were added to the general linear models as covariates of no interest.

Despite a lack of correlation between right pallidal size and causal awareness in HC, we conducted comparative shape analyses for this region. In HC there were no specific right pallidum vertices correlated with causal awareness, demonstrating a lack of a consistent region of relative atrophy within this group.

For comparison with the literature, DEP and HC subcortical volumes were compared. DEP had reduced volume relative to HC in the left pallidum, *t*(58)=2.9, *P*=0.005, left thalamus, *t*(58)=3.23, *P*=0.002 and right thalamus, *t*(58)=2.43, *P*=0.018 (see Supplementary Materials; Supplementary Table 1).

### Right dorsolateral pallidal surfaces connect with the striatum, ventral pallidum, dorsal thalamus and hippocampus

**Figure 5:**
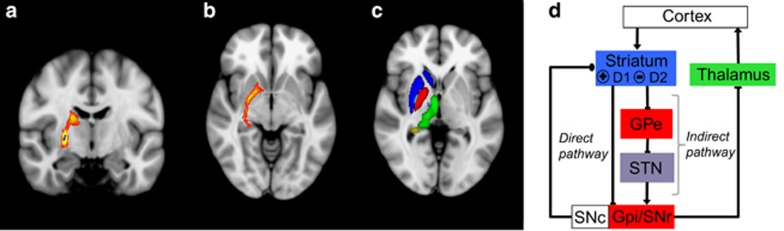
Probabilistic tractography. (**a**) Seed mask (blue) of the vertices significantly correlated with causal awareness. This region was primarily connected with the striatum, dorsal thalamus and (**b**) the hippocampus. (**c**) Regions of interest labeled on an axial slice: blue, striatum; red, pallidum; green, thalamus; purple, STN; yellow, hippocampus; light blue, GPe seed mask. (**d**) Schematic of the direct and indirect pathways within the basal ganglia. Arrows represent glutamatergic connections, flat ends represent GABAergic connections and round ends represent dopaminergic projections. GPe, globus pallidus externa; GPi, globus pallidus interna; SNc, substantia nigra pars compacta; SNr, substantia nigra pars reticulate; STN, subthalamic nucleus.

Figure 5 shows the group maps that resulted from running probabilistic tractography from right pallidal vertices that significantly correlated with causal awareness in DEP subjects with the top 50% of causal awareness (that is, individuals with normative volumes within the seed region). Tracts seeded from this mask revealed white matter connections with the striatum, dorsal thalamus and hippocampus. We found no gray matter regions correlating with causal awareness using VBM.

### Medication effects

*A t*-test comparison between medicated (*n*=32) and unmedicated participants (*n*=11) did not find any between-group differences in causal awareness or proportion of optimal choices. There were no correlations between unmedicated participants and subcortical sizes, although this may be due to the small sample size (*n*=9). In the medicated-only group, the correlation between causal awareness and right pallidal size remained significant, *r*=0.49, *P*=0.006.

## Discussion

Abnormalities within the cortico–striatal–pallidal–thalamic network have been widely reported in depression. Here, we investigated whether any structural differences within this circuit were associated with the level of causal awareness in young people with depression. Notably, volumetric analyses revealed a strong correlation, in the depressed sample only, between causal awareness and right pallidal size. Subsequent vertex-based shape analysis identified a localized correlation between reduced causal awareness and an inward position of surfaces on the right dorsolateral pallidum only, suggesting imbalance between the direct and indirect basal ganglia pathways. Probabilistic tractography revealed that these significantly correlated vertices were primarily connected with the striatum, dorsal thalamus and hippocampus.

Previous functional imaging in healthy subjects has shown that activity in the medial prefrontal cortex and a region of the associative striatum, the anterior caudate nucleus, are important during the encoding and retrieval of action-outcome contingencies and for exerting behavioral control over instrumental actions.^16, 17^ These regions are anatomically connected via a major cortico–basal ganglia circuit.^32, 33, 34^ Input to the associative striatum is then relayed to the principle striatal output nuclei, the substantia nigra pars reticulate and globus pallidus interna either directly, or indirectly, via the globus pallidus externa (GPe) and subthalamic nucleus. Medium spiny neurons in the direct pathway predominantly express dopamine D1 receptors, whereas those in the indirect pathway predominantly express D2 receptors, and these pathways are generally thought to promote and to inhibit basal ganglia output, respectively.^35, 36^

In our results, we found that the positive correlation between right pallidal volume and causal awareness was specifically due to inward location of surface vertices on the dorsolateral pallidum, a region that is part of the GPe. This finding is supported by a number of previous studies, reporting an association between D2 receptor-related activity and indirect pathway dysfunction in the cognitive control of action. For example, D2 receptor signaling has been linked to the updating of task-related working-memory representations,^37, 38^ and the loss of striatal D2 receptors with decreased cognitive flexibility.^39^ It has been acknowledged that alterations in dopamine transmission, particularly related to D2 receptor activity, may be an important contributor to cognitive impairments across multiple disorders.^40^ This raises the question of how indirect pathway dysfunction might produce cognitive deficits. One hypothesis^41^ proposes that the cortex represents multiple competing actions, whereas the basal ganglia selectively gate all but the most salient options^42^ through activity in the indirect pathway.^43^ In similar fashion, recent computational models of executive functions, such as working memory, suggest that the basal ganglia have a gate-keeping role for the flow of information into memory,^38, 44^ with activation in the direct pathway resulting in increased communication with the prefrontal cortex (PFC) and updating working memory, whereas activation in the indirect pathway restricts such modulation, thereby reducing interference with PFC function.

In this context it is important to note that, although reduced right GPe volume and changes in vertex location were related to changes in causal awareness in our depressed group, these correlations were not observed within the healthy group. This is possibly due to a floor effect, with individual causal judgments being relatively unimpaired in the latter group. In addition, right pallidal size did not differ between the groups. This could suggest that an imbalance between the direct and indirect pathways alters causal awareness, rather than reduced overall volume. By extension to the functions of the direct and indirect pathways, GPe–globus pallidus interna balance is similarly important for normal feedback to modulate cortical activity.^45^ This has important implications for other research using volumetric comparisons, as the overall size of the individual structures may not be the most informative metric. It is unclear why this finding should occur only in the right hemisphere; however, Shah *et al.*^21^ reported right fronto-striatal atrophy in subjects with treatment-resistant depression, which may point toward a particular subgroup of cognitively impaired individuals who are at a greater risk.

The GPe is centrally located within a number of basal ganglia feedforward and feedback circuits.^46^ Our probabilistic tractography data revealed that GPe surfaces related to causal awareness were primarily connected with the striatum, dorsal thalamus and hippocampus.

Notably, there were no subthalamic nucleus projections, suggesting that the affected circuits may involve feedback rather than feedforward connections. Although it is possible that our imaging resolution precluded the detection of tracts to the subthalamic nucleus, Mallet *et al.*^47^ have recently described a population of striatal projecting pallidal neurons that fit this profile, and that could be responsible for modulating the balance between direct and indirect pathways, a suggestion to be assessed in future studies.

In contrast to the typical finding of smaller subcortical volumes in depression,^23, 48^ there were no overall size reductions relative to controls in the present study. This is most likely due to the age of the cohort and their relatively short illness durations. Disease-related atrophy has been shown to increase with longer illness durations and later stage of illness.^23, 49^ Although Matsuo *et al.*^19^ previously found reduced right striatal volumes in adolescent depression, they used a medication-naive sample. Medication use may have normative effects on subcortical gray volumes,^21, 50^ although no report has specifically demonstrated that this occurs in the pallidum. Medication, specifically antidepressants, has variably been associated with changes in cognitive function,^51, 52^ and here our medication sub-analysis did not show any difference between medicated and non-medicated depressed subjects; however, this needs to be replicated with greater numbers, particularly as serotonin has been shown to suppress GABAergic inhibition in the GPe.^53^

Although we may have expected a correlation between gray matter volume in the medial prefrontal cortex and causal awareness, we did not find any evidence of this using VBM; however, the normalization process used in VBM can make the technique less sensitive to the subtle changes that we might expect in a youth cohort.^54^ Alternatively, despite the common assumption that most cognitive dysfunction is largely associated with altered PFC activity, Simpson *et al.*^55^ have postulated that basal ganglia dysfunction could in fact be driving this altered prefrontal activity. Indeed, decreasing activity in the mediodorsal thalamus, a relay structure between the striatum and the PFC, disrupts prefrontal-dependent cognitive behavior.^56^ As the current sample was at a relatively early stage in their course of illness, it is possible that GPe dysfunction is in fact the root cause of the problem, and that detectable prefrontal structural changes have not yet emerged.

Our behavioral data suggest that sensitivity to the causal consequences of specific actions is quite varied in young people with depression, and that this measure is not necessarily correlated with the severity of mood symptoms. Although the DEP group had reduced causal awareness on average relative to HCs, there were a number of unimpaired individuals. This was not unexpected; it has long been noted that the diagnostic criteria for major depressive disorder includes a broad range of symptomologies and aetiologies.^57^ The task that we have developed, however, may allow us to identify a functionally and biologically relevant subgroup of depressed individuals in which more targeted interventions could be applied. Increased cognitive impairments in depression lead to greater functional disability,^4^ which was supported by our finding that decreased causal awareness was associated with reduced social and occupational functioning. Further, social and occupational functioning was not necessarily reduced in those with higher depression severity. As cognitive impairment has been associated with higher rates of relapse into depressive episodes,^58^ early identification and treatment of functional deficits such as these may be critical for changing the trajectory of the illness. Furthermore, response to treatment may be more favorable in the earlier stages of illness at a time when behavioral and cognitive styles are less entrenched.^59^

In conclusion, volumetric reduction of the right dorsolateral pallidum of youths with depression was associated with changes in their ability to gauge the causal consequences of their actions. Impairments in this critical capacity are associated with decreases in social and occupational functioning, which likely increases disease burden and, as such, elucidating the etiology of this abnormality and further defining the role of cortico–striato–pallidal–thalamic circuits will help the development of new targets for cognitive treatments.

## Figures and Tables

**Table 1:** Demographic and clinical characteristics of participants

	*DEP (*n*=43)*	*HC (*n*=21)*	*t/X^2^ (*P*)*
*Demographics*			
Gender, *N* female (%)	34 (69%)	9 (56%)	0.93 (0.25)
Age, years	22.2±3.1	23.6±2.2	1.87 (0.07)
Education	13.2±2.0	14.2±2.5	1.55 (0.13)
Predicted IQ	104.6±7.5	106.6±8.5	0.85 (0.40)
			
*Symptoms and history*			
Age of onset (years)	13.7±3.4		
Duration of illness (years)	8.1±4.1		
HDRS	13.8±7.2	0.9±1.5	−11.0 (<0.001)**
SOFAS	63.1±10.3	91.4±2.6	10.55 (<0.001)**
			
*Medication*			
% Medicated	74.4		
% On antidepressants	57.1		
% On mood stabilizers/anticonvulsants	16.3		
% On antipsychotics	25.6		
% On anxiolytics	0.02		
			
*Motivation measures*			
Hunger (0–10)	5.9±2.2	5.8±1.8	−0.13 (0.90)
Food ratings (−5–+5)	2.1±1.9	2.4±1.7	0.36 (0.72)
Press rate (s^−1^)	0.71±0.2	0.73±0.2	0.39 (0.70)
			

Abbreviations: DEP, depression; HC, healthy control; HDRS, Hamilton Depression Rating Scale; IQ, intelligence quotient; SOFAS, Social and Occupational Functioning Assessment Scale.

NB: duration of illness indicates time since patient first experienced mental health problems, not time since diagnosis. **Denotes <0.001.

The data are represented as mean±s.d.
