# Supplementary material for: Right external globus pallidus changes are associated with altered causal awareness in youth with depression
Source: Transl Psychiatry. 2015 Oct 6;5(10):e653–. doi: 10.1038/tp.2015.148 (PMC4930125; doi:10.1038/tp.2015.148)
Supplement: Supplementary Information [file tp2015148x1.docx]

***Methods***

*Task Instructions*

In this experiment, you will press buttons to earn food rewards. Place the pointer finger of your left hand over the left yellow key and the pointer finger of your right hand over the right yellow key (*demonstrate*). You will see two yellow boxes in the middle of the screen – these correspond to the yellow keys that you have your fingers on. During the task you should press these buttons that your fingers are on. You will see a gray circle appear, or **sometimes** you will see a picture of a barbeque shape or M&M appear. When you see these pictures, it means that you have earned that food, and there will be a running tally on the bottom of the screen telling you how many MnMs and BBQ shapes you have accumulated. You should press the buttons in a way that will earn you as many points as you can. You should also pay attention to how likely it is that a particular button will earn you a particular reward. At the end of each block a screen will appear asking, “How likely was it that pressing the left [or right] earned you an M&M [or barbeque shape]?”. Use the mouse to record your answer, clicking on any number from 0, not at all likely, to 10, extremely likely. At the end of this experiment, you will receive the food you have earned.

*Image acquisition and analysis*

Participants underwent structural MRI scanning using a 3-Tesla GE sMR750 MRI scanner at the Brain and Mind Research Institute, Camperdown, NSW Australia. Images were acquired using a customized MP-RAGE 3D T1-weighted sequence to resolve anatomy at high resolution (0.9mm isotropic resolution) with the following parameters: TR = 7.2ms; TE = 2.8ms; flip angle = 10°; coronal orientation; FOV 230 mm3; matrix of 256 x 256, 196 total slices. Next whole brain diffusion-weighted images were also acquired using an echo planar imaging sequence with the following parameters: TR=8250ms; TE=85ms; number of slices=55 thickness=2mm-thick axial slices; matrix size, 128 x 128; in-plane resolution, 1.8 x 1.8mm2;

69 gradient directions. Eight images without gradient loading (B0 s.mm-2) were acquired prior to the acquisition of 69 images with uniform gradient loading (B0 = 1000s.mm-2).

*Voxel-based morphometry*

Two T1-weighted structural scans obtained from a single scanning session were averaged for each individual using FSLmaths to increase signal-to-noise ratio. We carried out an unbiased optimised VBM protocol 34 beginning with brain extraction. FSL BET 35 was applied to remove non-brain material, before all T1-weighted images were transformed into standard space using a limited degrees-of-freedom non-linear model to ensure spatial alignment and images were corrected for non-uniformity. The FAST4 tool 36 was then applied to carry out tissue- type segmentation. The segmented grey matter partial volume images were aligned into MNI

standard space by applying the affine registration tool FLIRT 37 and nonlinear registration FNIRT methods 38, which use a B-spline representation of the registration warp field. A study- specific averaged template was created, to which grey matter partial volume images were re- registered, and these images were then modulated to correct for Jacobian warping. Visual inspection was used to ensure the quality of brain image extraction, segmentation, and registration for each structural image. Segmented images were smoothed using a Gaussian kernal with 3 mm standard deviation (FWHM 7.05 mm). Correlations between causal awareness and grey matter volumes were assessed using permutation-based GLM, both with and without covariance for depression severity. Group differences were also assessed using F statistics co-varied for age and education.

*Volumetrics*

The semi automated FIRST 27 routine was used to segment the following: bilateral caudate nucleus, putamen, pallidum and thalamus. All segmentations were visually inspected to ensure that there were no gross registration or segmentation errors. Tissue-type segmentation carried out using FAST4 was used to calculate intracranial volumes (ICV), which were used to correct for differences in head size. The aforementioned sub-cortical region volumes were then corrected for ICV variation so as to provide a common space for cross- sectional morphometric comparisons. All statistical analyses were conducted using IBM SPSS Statistics. Pearson correlations were conducted within each group between causal awareness and sub-cortical volumes, and clinical measure scores. Volumetric differences between

groups were also assessed.

*Shape Analysis*

Localized shape differences in subcortical regions were also examined in cases where volumetric changes were significantly correlated to the task performance. FIRST created a surface mesh for each subcortical structure in each subject, which was reconstructed in MNI space to normalize for inter-individual head size differences. Pose (rotation and translation) was removed by minimizing the sum of squares difference between the corresponding vertices of a subject’s surface and the mean surface. Correlations between causal awareness

and sub-cortical region shape were assessed within the depressed group on a per-vertex basis using permutation-based GLM, both with and without co-variance for depression severity. Group differences were also assessed on a per-vertex basis using F statistics co-varied for age and education. The directionality of significant F tests was investigated using t tests.

*Diffusion Tensor Imaging Preprocessing and Qualitative Probabilistic Tractography*

Data was firstly eddy-current corrected using FMRIB Diffusion Toolbox to align all images to a reference b0 image and linearly transform them 39, brains were extracted, and diffusion tensors fitted. Diffusion probabilistic tractography was then performed using the FDT Diffusion Toolbox 40,41. For each subject, tractography was run from a seed mask of vertices significantly correlated with causal awareness within the depressed group.

Tractography was performed from every voxel within the seed mask to build up a connectivity distribution. We fitted a three-fiber orientation diffusion model 39 to estimate probability distributions on the direction of fiber populations at each brain voxel in the diffusion space of each subject. To interpret the probabilistic tractography in standard space, we used standard-to-diffusion matrices and the corresponding inversed matrices. We generated 5000 samples from each seed voxel with a curvature threshold of 0.2 and no waypoint or termination masks. Tracking was performed in diffusion space, with results transformed back to MNI space to produce a whole-brain image for each seed region. To visualize tracts efferent and afferent to the seed mask, individual participant 3D files were thresholded to the top 10% of tracts and binarized, before being concatenated into a 4D file.

**Supplementary Table 1. ICV-corrected volumetric data of ROIs; mean** ± **SEM (mm3) DEP (*n=44*) HC (*n=16*)**

**Left Hemisphere** Caudate 3766 ± 59 3788 ± 80

|  | Putamen | 4815 ± | 55 | 4986 ± | 102 |
| --- | --- | --- | --- | --- | --- |
|  | Pallidum | 1660 ± | 15 | 1706 ± | 19 |
|  | Thalamus | 8052 ± | 61 | 8236 ± | 122 |
| **Right Hemisphere** | Caudate | 3990 ± | 64 | 4008 ± | 117 |
|  | Putamen | 4790 ± | 90 | 4996 ± | 119 |
|  | Pallidum | 1670 ± | 17 | 1676 ± | 30 |
|  | Thalamus | 7923 ± | 62 | 7978 ± | 89 |

Comparison of the ICV corrected volumes of the regions of interest showed no significant differences in volumes.
